# Tyrosine kinase inhibitors of Ripk2 attenuate bacterial cell wall-mediated lipolysis, inflammation and dysglycemia

**DOI:** 10.1038/s41598-017-01822-0

**Published:** 2017-05-08

**Authors:** Brittany M. Duggan, Kevin P. Foley, Brandyn D. Henriksbo, Joseph F. Cavallari, Akhilesh K. Tamrakar, Jonathan D. Schertzer

**Affiliations:** Department of Biochemistry and Biomedical Sciences and Department of Pediatrics, McMaster University, Hamilton, ON L8N 3Z5 Canada

## Abstract

Inflammation underpins aspects of insulin resistance and dysglycemia. Microbiota-derived cell wall components such as muropeptides or endotoxin can trigger changes in host immunity and metabolism. Specific peptidoglycan motifs promote metabolic tissue inflammation, lipolysis and insulin resistance via Nucleotide-binding oligomerization domain-containing protein 1 (Nod1). Receptor-interacting serine/threonine-protein kinase 2 (Ripk2) mediates Nod1-induced immunity, but the role of Ripk2 in metabolism is ill-defined. We hypothesized that Ripk2 was required for Nod1-mediated inflammation, lipolysis and dysglycemia. This is relevant because certain tyrosine kinase inhibitors (TKIs) inhibit Ripk2 and there is clinical evidence of TKIs lowering inflammation and blood glucose. Here, we showed that only a subset of TKIs known to inhibit Ripk2 attenuated Nod1 ligand-mediated adipocyte lipolysis. TKIs that inhibit Ripk2 decreased cytokine responses induced by Nod1-activating peptidoglycan, but not endotoxin in both metabolic and immune cells. Pre-treatment of adipocytes or macrophages with the TKI gefitinib inhibited Nod1-induced Cxcl1 and Il-6 secretion. Furthermore, treatment of mice with gefitinib prevented Nod1-induced glucose intolerance *in vivo*. Ripk2 was required for these effects on inflammation and metabolism, since Nod1-mediated cytokine and blood glucose changes were absent in Ripk2^−/−^ mice. Our data show that specific TKIs used in cancer also inhibit Nod1-Ripk2 immunometabolism responses indicative of metabolic disease.

## Introduction

Tyrosine kinase inhibitors (TKIs) that interfere with growth factor receptor or proto-oncogene pathways are extensively used in cancer treatment. There are more than 20 TKIs currently approved for clinical use and many more in the pipeline^1^. Clinical observations have documented significant reductions in blood glucose, and reduced dependence on insulin and other anti-hyperglycemic medications in diabetic cancer patients receiving TKI therapy^2–5^. Certain TKIs have been investigated for the treatment of pre-diabetes and diabetes and preliminary animal studies lend support to the anti-hyperglycemic effects of TKIs. For example, PD153035 (a TKI designed against epidermal growth factor receptor (EGFR)) improves both glucose tolerance and insulin action in diet-induced obese mice^6^. The TKIs imatinib and sunitinib have been shown to reduce insulin resistance in multiple obese rodent models^7–10^. Despite consistent and convincing clinical and animal data supporting a role for TKIs in blood glucose regulation, the mechanism underlying these effects remains largely unknown and it is not clear what target(s) of various TKIs are responsible for propagating their glucose-lowering effects.

Chronic inflammation is now a well-established component in the etiology of obesity-related insulin resistance, dysglycemia, type 2 diabetes and cardiovascular disease^11, 12^. Pattern recognition receptors (PRRs) of the innate immune system, such as toll-like receptors (Tlrs) and nucleotide oligomerization domain-containing proteins (Nods) can propagate obesity-related inflammation and metabolic dysfunction^13–16^. Triggers of inflammation during obesity or metabolic disease include pathogen-associated molecular pattern (PAMPS), such as bacterial-derived endotoxin and bacterial cell wall-derived peptidoglycan. For example, a low-level increase in lipopolysaccharide (LPS) can promote dysglycemia via Tlr4^17^. Direct activation of Nod1 with specific bacterial cell wall muropeptides (i.e. peptidoglycan) can induce inflammation, augment adipocyte lipolysis, and promote glucose intolerance and insulin resistance^13, 15, 18^.

Nod1 is an intracellular sensor of meso-diaminopimelic acid (DAP)-containing peptidoglycan (PGN), primarily derived from the cell wall of Gram negative bacteria^19^. Nod1 activation by meso-DAP containing PGN induces pro-inflammatory signalling by recruitment and activation of receptor-interacting protein kinase 2 (Ripk2)^20^. Upon activation, Ripk2 undergoes autophosphorylation and polyubiquitylation, which promotes activation of mitogen-activated protein kinase (MAPK) and NF-κB and signalling pathways^21^. Ripk2 is known to propagate inflammatory signals in response to Nod1 activation, but the role of Ripk2 in metabolic perturbations downstream of Nod1 has yet to be established.

Interestingly, Ripk2 has emerged as a target of certain tyrosine kinase inhibitors (TKIs)^22^. For example, the TKIs gefitinib and erlotinib have been reported to inhibit Ripk2 with an equal half-maximal inhibitory concentration (IC_50_) compared to their intended target protein EGFR^23, 24^. Based on the relation between acute Nod1 signalling and prominent features of metabolic disease, and the ability of TKIs to inhibit Ripk2, we hypothesized that: (1) Ripk2 was required for Nod1-mediated immunometabolism effects, (2) TKIs that inhibit Ripk2 would lower Nod1-ligand induced lipolysis, inflammation and dysglycemia. Using TKIs known to inhibit ALK, BTK, BCR-Abl, c-Kit, c-Met, EGFR, JAK1/2, PDGFR, RAF kinases (b-RAF and c-RAF), RET, Src or VEGFR, we showed that inhibition of Ripk2 defines that ability of certain TKIs to attenuate bacterial cell wall-triggered inflammation and metabolic defects. These results characterize a potential mechanism contributing to TKI-mediated improvements in blood glucose control.

## Results

### Selected TKIs inhibit bacterial cell wall-induced lipolysis

**Figure 1:**
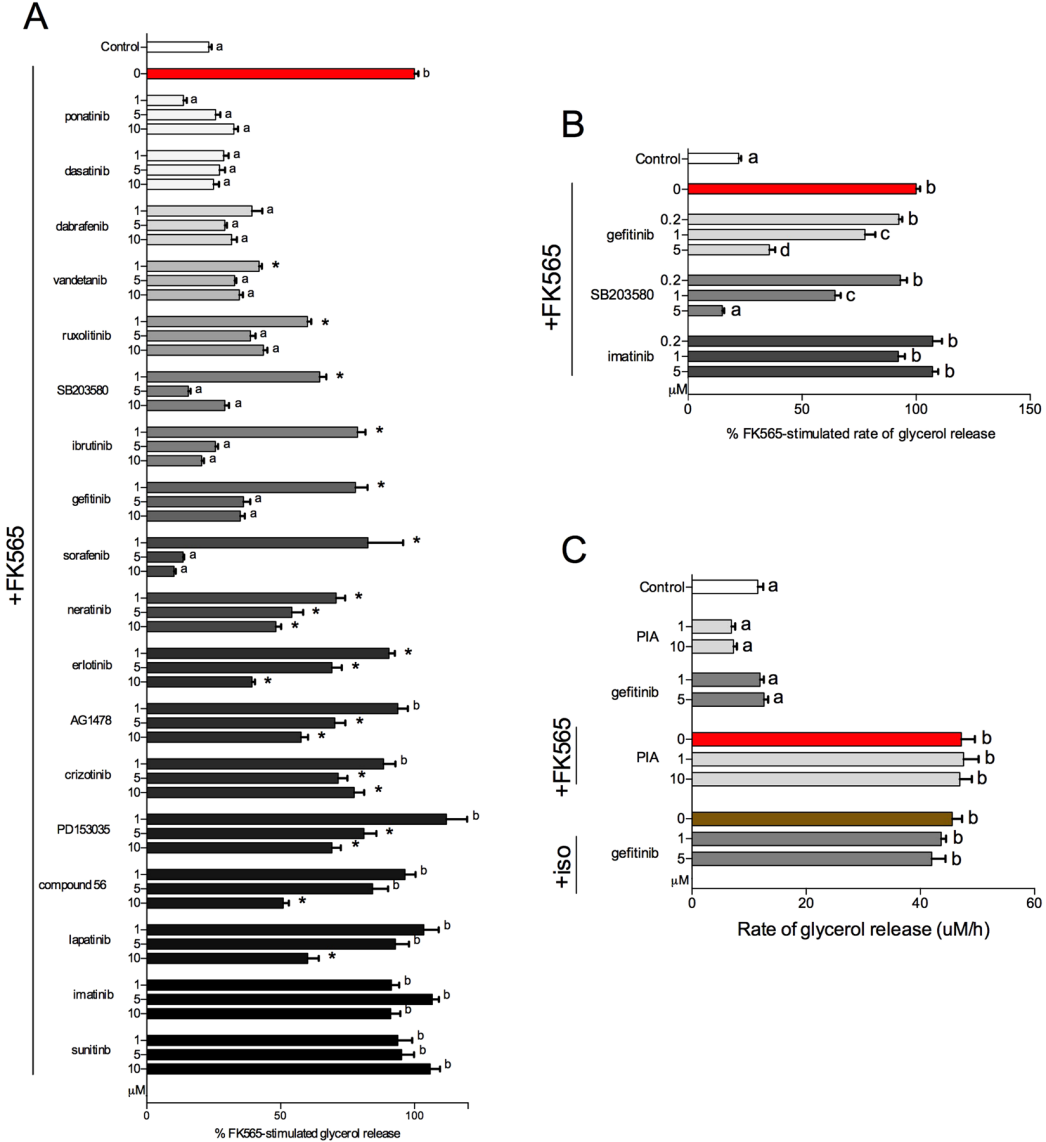
Selected TKIs inhibit bacterial cell wall-mediated lipolysis in adipocytes. Relative levels of glycerol released from 3T3-L1 adipocytes after stimulation with the Nod1 ligand FK565 (10 μg/mL) for 48 h and pre-incubated for 1 h with 1, 5 or 10 μM of various TKIs (**A**) n = 6–69. Rate of glycerol release over 48 h in 3T3-L1 adipocytes pre-incubated for 1 h with 0.2, 1 or 5 μM gefitinib, SB203580, and imatinib (**B**) n = 8–49. Rate of glycerol release over 48 h in 3T3-L1 adipocytes pre-incubated for 1 h with PIA (1 or 10 μM) and stimulated with FK565 (10 μg/mL), or pre-incubated with gefitinib (1 or 5 μM) and stimulated with isoproterenol (2 μM) respectively (**C**) n = 6–10. Values are mean ± SEM. Conditions with different letters (a, b, c) denote a statistical difference compared to all other conditions without the same letter (P < 0.05). *Denotes partial inhibition that is statistically lower than FK565-stimulated glycerol release, but significantly higher than basal glycerol release for a given TKI (p < 0.05).

We previously reported that activation of Nod1 with 10 μg/mL of the muramyl tetrapeptide, heptanoyl-γ-D-glutamyl-(L)-meso-diaminopimelyl-(D)-alanine (FK565) augmented lipolysis in 3T3-L1 adipocytes and murine adipose tissue in a Nod1-dependent manner^13^. Here, we assessed if different types of TKIs altered Nod1-induced lipolysis. Our results show that TKIs (with diverse intended target proteins) inhibited Nod1-mediated lipolysis in a dose-dependent manner (Fig. 1A,B). FK565-stimulated glycerol release was lower in 3T3-L1 adipocytes pre-incubated with TKIs ponatinib, dasatinib, dafabranib, vandetanib, ruxolitinib, SB203580, ibrutinib, gefitinib, sorafenib, neratinib, erlotinib, at 1–10 μM (Fig. 1A). FK565-sitmulated glycerol release was lower in 3T3-L1 adipocytes pre-incubated with AG1478, crizotinib and PD153035, at 5–10 μM. Compound 56 and lapatinib at 10 μM inhibited FK565-stimulated lipolysis (Fig. 1A). However, imatinib and sunitinib, which do not have reported inhibitory activities against Ripk2^25^, did not alter FK565-stimuluated glycerol release (Fig. 1A).

We next determined the FK565-stimluated rate of glycerol release between 0 and 48 h with pre-incubation of gefitinib, SB203580 (a known Ripk2 inhibitor) and imatinib, a TKI which did not display inhibitory activity against Nod1-mediated lipolysis. Only 1 or 5 μM gefitinib and 1 or 5 μM SB203580 dose dependently attenuated the increased rate of glycerol release induced by FK565 (Fig. 1B). Imatinib did not alter the rate of FK565-induced glycerol release over 48 h (Fig. 1B). Gefitinib (1–5 μM) reduced FK565-stimulated glycerol levels in the media at both 24 and 48 h (see Supplementary Fig. S1). Induction of lipolysis by FK565 was coincident with decreases in several molecular markers of lipid oxidation, transport and storage at 24 h and 48 h. Pre-treatment with gefitinib partially or completely restored the majority of these changes in transcript levels (see Supplementary Fig. S1). Because of the robust effects that TKIs had on Nod1-mediated lipolysis, we sought to differentiate the effects of TKIs on inflammatory-induced lipolysis versus hormonal/adrenergic lipolytic programs. We tested effects of (-)-N^6^-(2-Phenylisopropyl)adenosine (PIA) on glycerol release from adipocytes treated with Nod1 ligand. PIA is an analogue of adenosine that is a well-established inhibitor of catecholamine-induced lipolysis^26–28^. PIA (1–10 μM) pre-treatment did not alter FK565-stimulated lipolysis (Fig. 1C). Furthermore, we tested the ability of the TKI gefitinib to attenuate isoproterenol-induced lipolysis. Gefitinib (1 or 5 μM), had no effect on isoproterenol-induce lipolysis. Our results show that distinct pathways are engaged by Nod1-mediated inflammatory-related lipolysis versus adrenergic or hormonal augmentation of lipolysis (Fig. 1C).

### Selected TKIs inhibit bacterial cell wall-induced, but not endotoxin-induced cytokine responses in metabolic and immune cells

**Figure 2:**
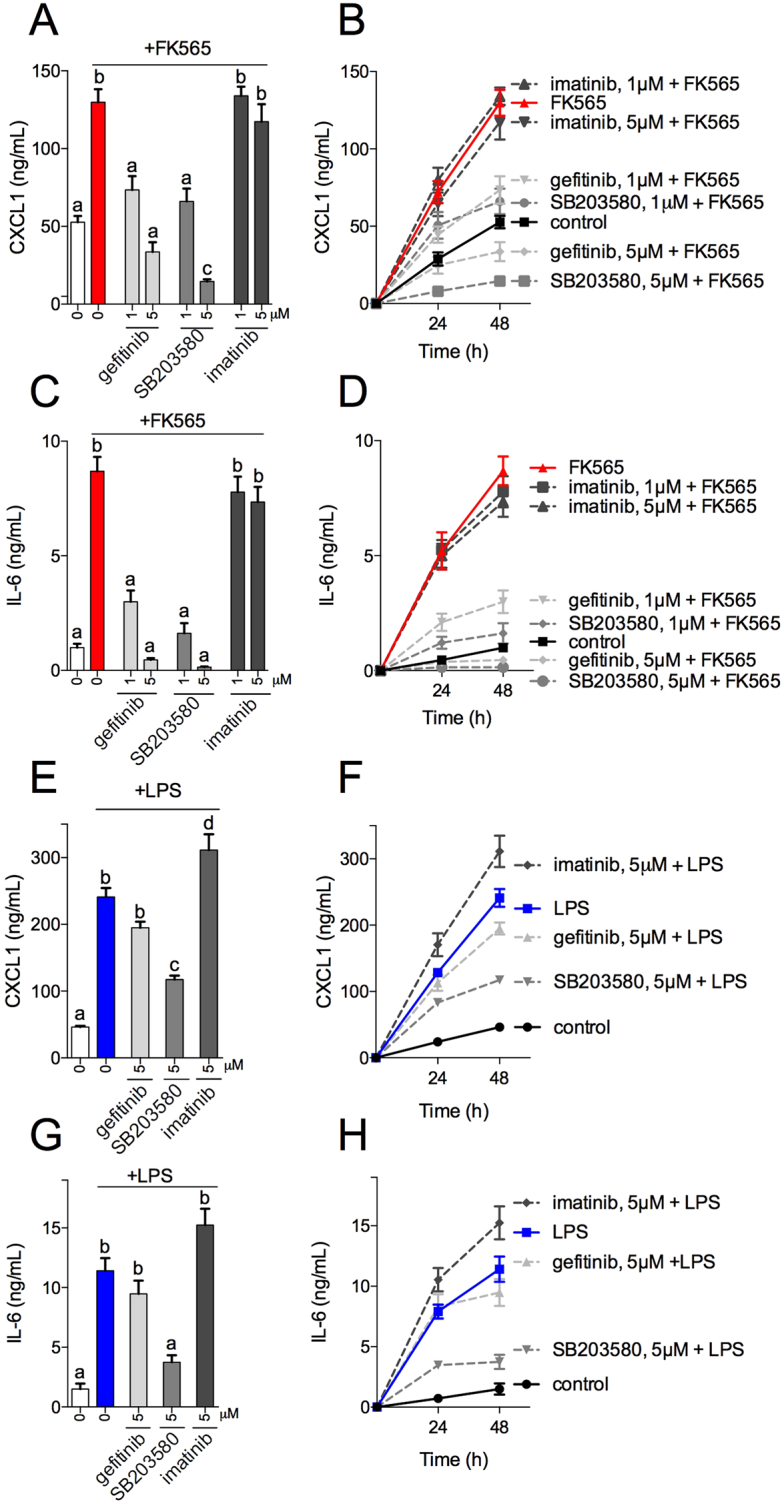
Selected TKIs inhibit cytokine responses in adipocytes induced by bacterial cell wall-mediated components, but not endotoxin. Levels of Cxcl1 released from 3T3-L1 adipocytes after stimulation with the Nod1 ligand FK565 (10 μg/mL) for 48 h and pre-incubated for 1 h with 1 or 5 μM of the TKIs gefitinib, SB203580 or imatinib (**A**) n = 6. Time course of FK565-stimulated Cxcl1 secretion in 3T3-L1 adipocytes (**B**) n = 6. Levels of Il6 released from 3T3-L1 adipocytes after stimulation with the Nod1 ligand FK565 (10 μg/mL) for 48 h and pre-incubated for 1 h with 1 or 5 μM of the TKIs gefitinib, SB203580 or imatinib (**C**) n = 5. Time course of FK565-stimulated Il6 secretion in 3T3-L1 adipocytes (**D**) n = 5. Levels of Cxcl1 released from 3T3-L1 adipocytes after stimulation with the Tlr4 ligand LPS (0.5 μg/mL) for 48 h and pre-incubated for 1 h with 1 or 5 μM of the TKIs gefitinib, SB203580 or imatinib (**E**) n = 6. Time course of LPS-stimulated Cxcl1 secretion in 3T3-L1 adipocytes (**F**) n = 6. Levels of Il6 released from 3T3-L1 adipocytes after stimulation with the Tlr4 ligand LPS (0.5 μg/mL) for 48 h and pre-incubated for 1 h with 1 or 5 μM of the TKIs gefitinib, SB203580 or imatinib (**G**) n = 5. Time course of LPS-stimulated Il-6 secretion in 3T3-L1 adipocytes (**H**) n = 5. Values are mean ± SEM. Different letters assigned to each condition (a, b, c) denote statistical differences between groups (p < 0.05).

We next determined if certain TKIs also attenuated Nod1-mediated inflammatory mediators in 3T3-L1 adipocytes and bone marrow derived macrophages (BMDM). We used secretion of the chemokine Cxcl1 and cytokine Il6 as pro-inflammatory indicators of Nod1 signalling, as previously published^15^. Our results show that pre-incubation with 1–5 μM gefitinib or 1–5 μM SB203580, but not 1–5 μM imatinib significantly attenuated FK565-induced Cxcl1 and Il6 release in a dose-dependent manner in adipocytes (Fig. 2A–D). In order to assess specificity of Ripk2 inhibitors for different components of the bacterial cell wall, we next tested these selected TKIs on LPS-mediated Cxcl1 and Il6 secretion. Our results show that 5 μM gefitinib or 5 μM imatinib did not alter LPS-induced secretion of Cxcl1 or Il6 in 3T3-L1 adipocytes (Fig. 2E–H). However, 5 μM SB203580 significantly lowered LPS-induced Cxcl1 and Il6 concentration in 3T3-L1 adipocytes (Fig. 2E–H). These results show that gefitinib inhibits Nod1-ligand, but not LPS-induced inflammation in adipocytes.

### Gefitinib attenuates bacterial cell wall-mediated insulin resistance in adipocytes

**Figure 3:**
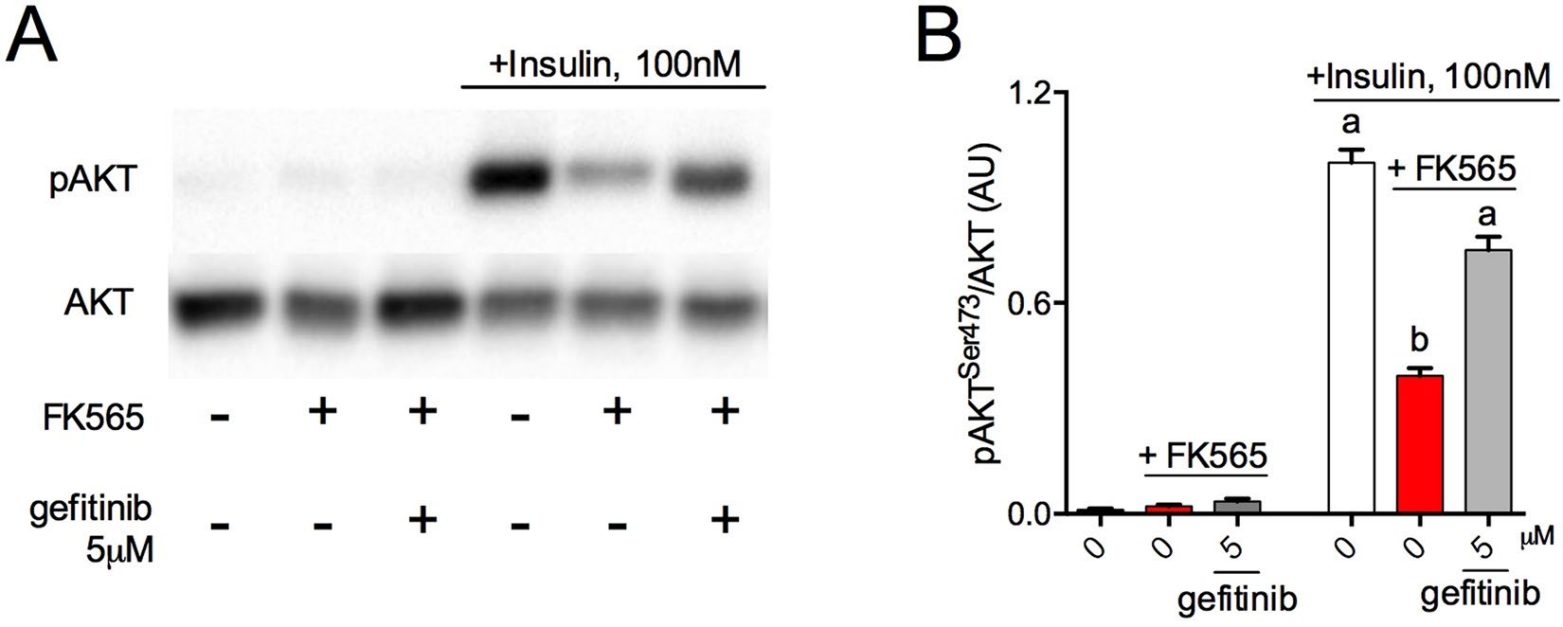
TKI gefitinib attenuates bacterial cell wall-mediated insulin resistance in adipocytes. Representative immunoblots (**A**) and quantification (**B**) of basal (i.e. no insulin) and 100 nM insulin-stimulated pAKT (Ser473) relative to total AKT in 3T3-L1 adipocytes after 1 h pre-incubation with gefitinib (5 μM) and treatment with FK565 (10 μg/mL) for 48 h. Quantitative comparison was conducted between samples from 4 different blots derived from the same experiment and processed in parallel. (n = 8 total (n = 2/condition per blot). Cropped images are displayed and tull images of all immunoblots are presented in Supplementary Fig. 3. Values are mean ± SEM. Different letters assigned to each condition (a, b) denote statistical differences between groups (p < 0.05).

Because of the recognized contributions of dyslipidemia and inflammation to insulin resistance, we sought to test if TKI-mediated inhibition of RIPK2 could attenuate NOD1-induced impairments in insulin signalling in adipocytes. We have previously shown that FK565 impaired insulin signalling in primary hepatocytes via Nod1^15^. Here, we demonstrate that stimulation of 3T3-L1 adipocytes with FK565 impairs the ability of insulin to phosphorylate Akt at serine 473, and that pre-incubation with 5 μM gefitinib completely restores this aspect of insulin signalling (Fig. 3A,B).

### Ripk2 is required for macrophage inflammation induced by bacterial cell wall muropeptides, but not endotoxin

**Figure 4:**
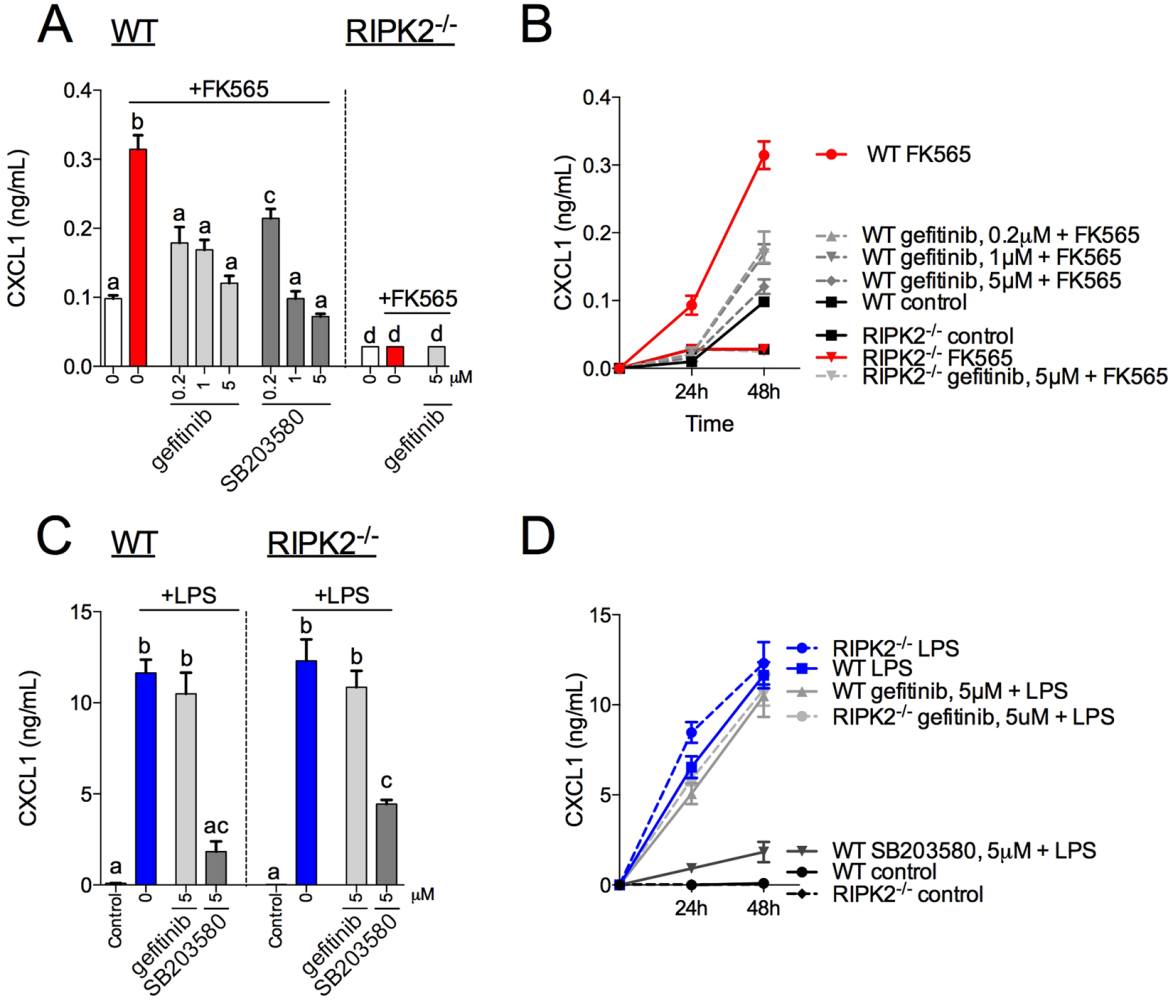
Selected TKIs inhibit RIPK2-mediated cytokine responses in macrophages induced by bacterial cell wall components, but not endotoxin. Levels of Cxcl1 released from WT (left panel) or RIPK2^−/−^ (right panel) bone marrow-derived macrophages (BMDMs) after stimulation with the Nod1 ligand FK565 (10 μg/mL) for 48 h and pre-incubated for 1 h with 0.2, 1 or 5 μM gefitinib or SB203580 (**A**) n = 4–12. Time course of FK565-stimulated Cxcl1 secretion in BMDMs (**B**) n = 4–12. Levels of Cxcl1 released from WT (left panel) or RIPK2^−/−^ (right panel) BMDMs after stimulation with the Tlr4 ligand LPS (0.5 μg/mL) for 48 h and pre-incubated for 1 h with 0.2, 1 or 5 μM gefitinib or SB203580 (**C**) n = 4–12. Time course of LPS-stimulated Cxcl1 secretion in BMDMs (**D**) n = 4–12. Values are mean ± SEM. Different letters assigned to each condition (a, b, c) denote statistical differences between groups (p < 0.05).

We next showed that specific TKIs attenuated inflammation from distinct bacterial ligands in primary macrophages. Gefitinib lowered FK565-induced, but not LPS-induced Cxcl1 secretion from BMDMs (Fig. 4A–D). BMDMs did not secrete a detectable level of Il6 in response to FK565 (see Supplementary Fig. S2). We confirmed that Ripk2 is required for Nod1-mediated inflammation by showing that BMDMs from RIPK2^−/−^ mice were refractory to FK565-induced Cxcl1 secretion (Fig. 4A, right panel). However, LPS-mediated cytokine responses were intact in BMDMs from Ripk2^−/−^ mice (Fig. 4C,D). LPS significantly increased Cxcl1 secretion in BMDMs derived from Ripk2^−/−^ mice and consistent with results from wild type (WT) macrophages, gefitinib did not alter LPS-induced Cxcl1 secretion in BMDMs from Ripk2^−/−^ mice (Fig. 4C,D).

### Gefitinib inhibits NF-κB activity induced by bacterial cell wall muropeptides, but not endotoxin

**Figure 5:**
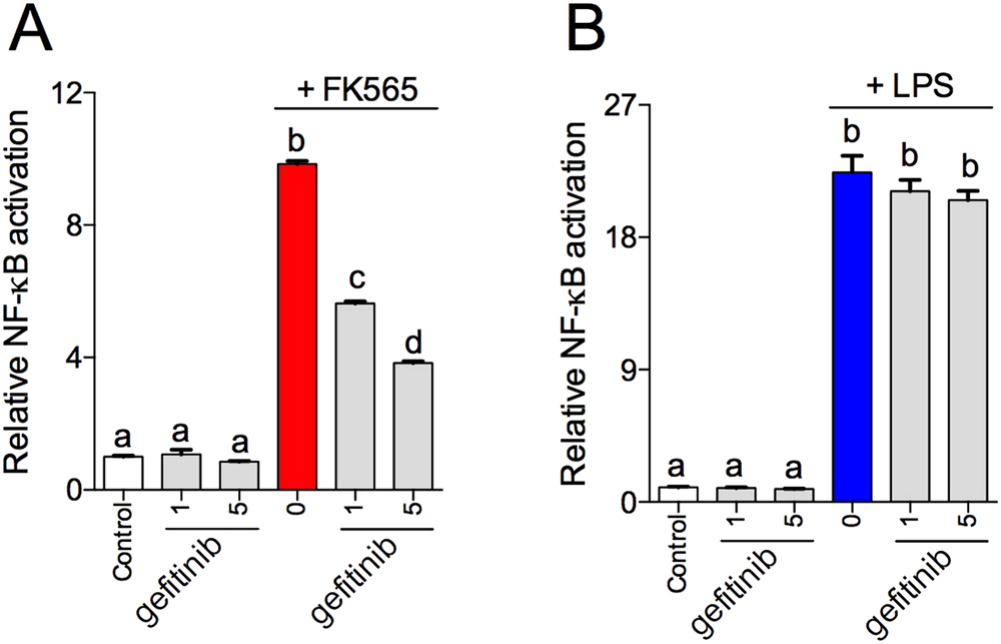
TKI gefitinib inhibits NF-κB activity induced by bacterial cell wall components, but not endotoxin. Relative NF-κB activity in HEK293 cells stably expressing Nod1, pre-incubated with gefitinib (1 or 5 μM) and stimulated with FK565 (10 μg/mL) (**A**), or in HEK293 cells stably expressing Tlr4, pre-incubated with gefitinib (1 or 5 μM) and stimulated with LPS (0.5 μg/mL) (**B**) n = 12. Values are mean ± SEM. Different letters assigned to each condition (a, b, c) denote statistical differences between groups (p < 0.05).

We next determined if gefitinib influenced bacterial ligand-specific inflammatory signals downstream of Ripk2. We assessed FK565- versus LPS-induced NF-κB activity using HEK293 cells stably over-expressing Nod1 or Tlr4. Consistent with cytokine secretion in adipocytes and macrophages, we find that treatment of HEK-Nod1 cells with FK565 (10 μg/mL) increases NF-κB activation, which is attenuated in a dose-dependent manner by pre-incubation with gefitinib at 1 and 5 μM (Fig. 5A). Conversely, gefitinib has no effect on NF-κB activation in response to LPS-induced Tlr4 activation in HEK-Tlr4 cells (Fig. 5B).

### Gefitinib inhibits Ripk2-mediated bacterial cell wall-induced inflammation and dysglycemia *in vivo*

**Figure 6:**
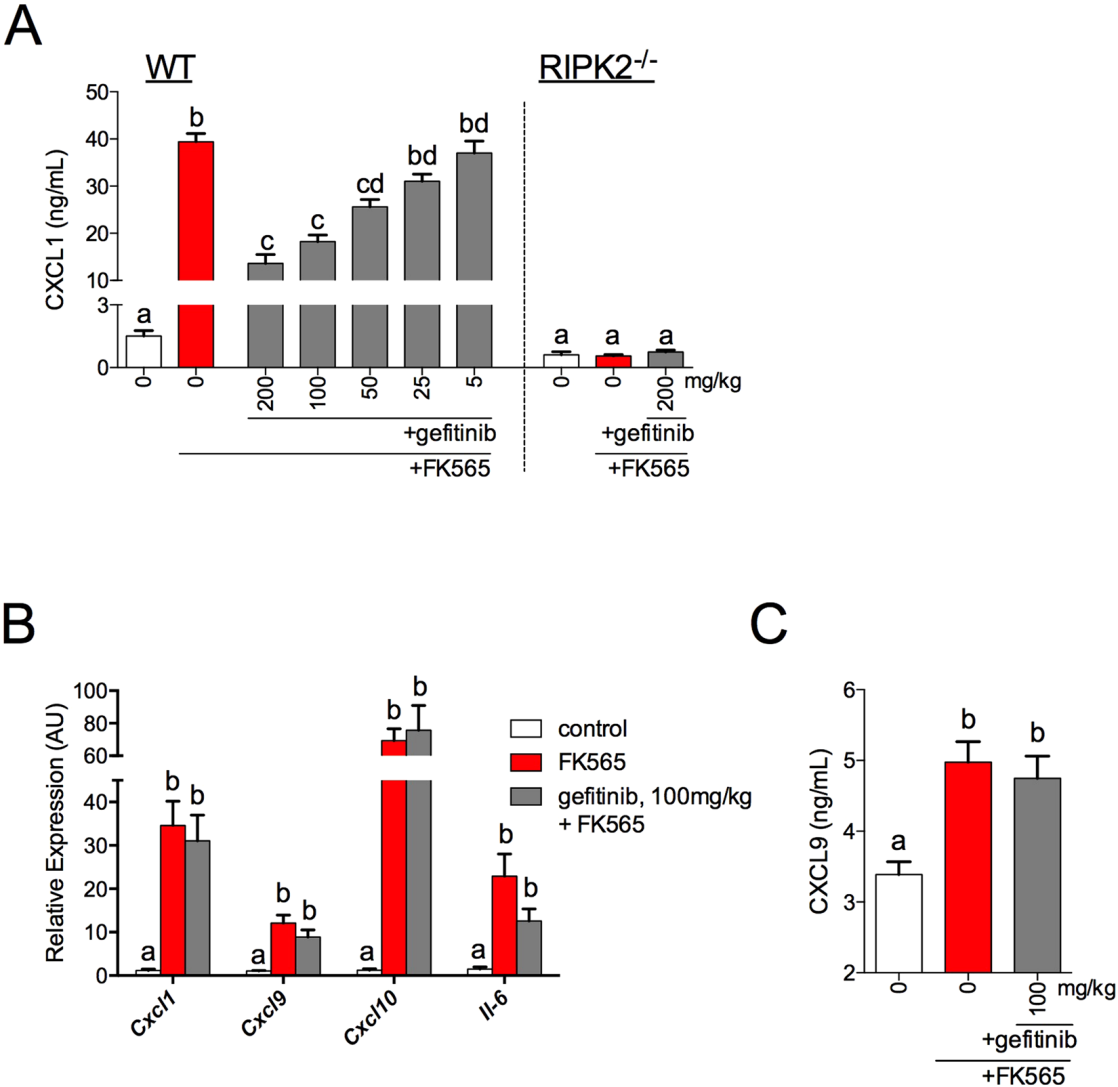
TKI gefitinib inhibits RIPK2-mediated inflammation *in vivo*. Cxcl1 secretion 2 h after injection with the Nod1 ligand FK565 (10 μg, *i*.*p*.) in WT or RIPK2^−/−^ mice that were pre-treated with various doses of gefitinib (5–200 mg/kg) or methylcellulose vehicle for 4 days (**A**) n = 4–60 per group. Transcript analysis of Cxcl1, Cxcl9, Cxcl10 and Il-6 in adipose tissue 6 h after injection of FK565 (10 μg, *i*.*p*.) in mice pre-treated with 100 mg/kg gefitinib or vehicle (**B**) n = 7–9. Protein levels of Cxcl9 in adipose tissue tissue 6 h after injection of FK565 (10 μg, *i*.*p*.) in mice pre-treated with 100 mg/kg gefitinib or vehicle (**C**) n = 8–9. Values are mean ± SEM. Different letters assigned to each condition (a, b, c) denote statistical differences between groups (p < 0.05).

In order to extend these cell-based results, mice were administered gefitinib (5–200 mg/kg/day) for 4 days, prior to injecting the Nod1 ligand FK565 (10 ug, *i*.*p*.). Our results show that treatment with gefitinib at doses equal or greater than 50 mg/kg attenuated Nod1-mediated Cxcl1 protein levels in the circulation of mice in a dose-dependent manner (Fig. 6A, left panel). We also confirmed that FK565 did not increase Cxcl1 serum levels in Ripk2^−/−^ mice (Fig. 6A, right panel). These *in vivo* cytokine results are based on a previous assessment of the time-course of serum Cxcl1 levels, where peak serum cytokines levels occurred at 2 h after *i*.*p*. injection of FK565 in WT mice (data not shown). To distinguish between systemic and adipose-specific inflammation, we assessed transcript levels of several inflammatory genes (Cxcl1, Cxcl9, Cxcl10, Il-6) in adipose tissue. Transcript levels of all of these inflammatory markers were increased by FK565 in the adipose tissue, but were not altered by pre-treatment of mice with 100 mg/kg gefitinib (Fig. 6B). Furthermore, protein levels of Cxcl9 were also increased by FK565 in the adipose tissue, but Cxcl9 was not altered by pre-treatment of mice with 100 mg/kg gefitinib (Fig. 6C).

**Figure 7:**
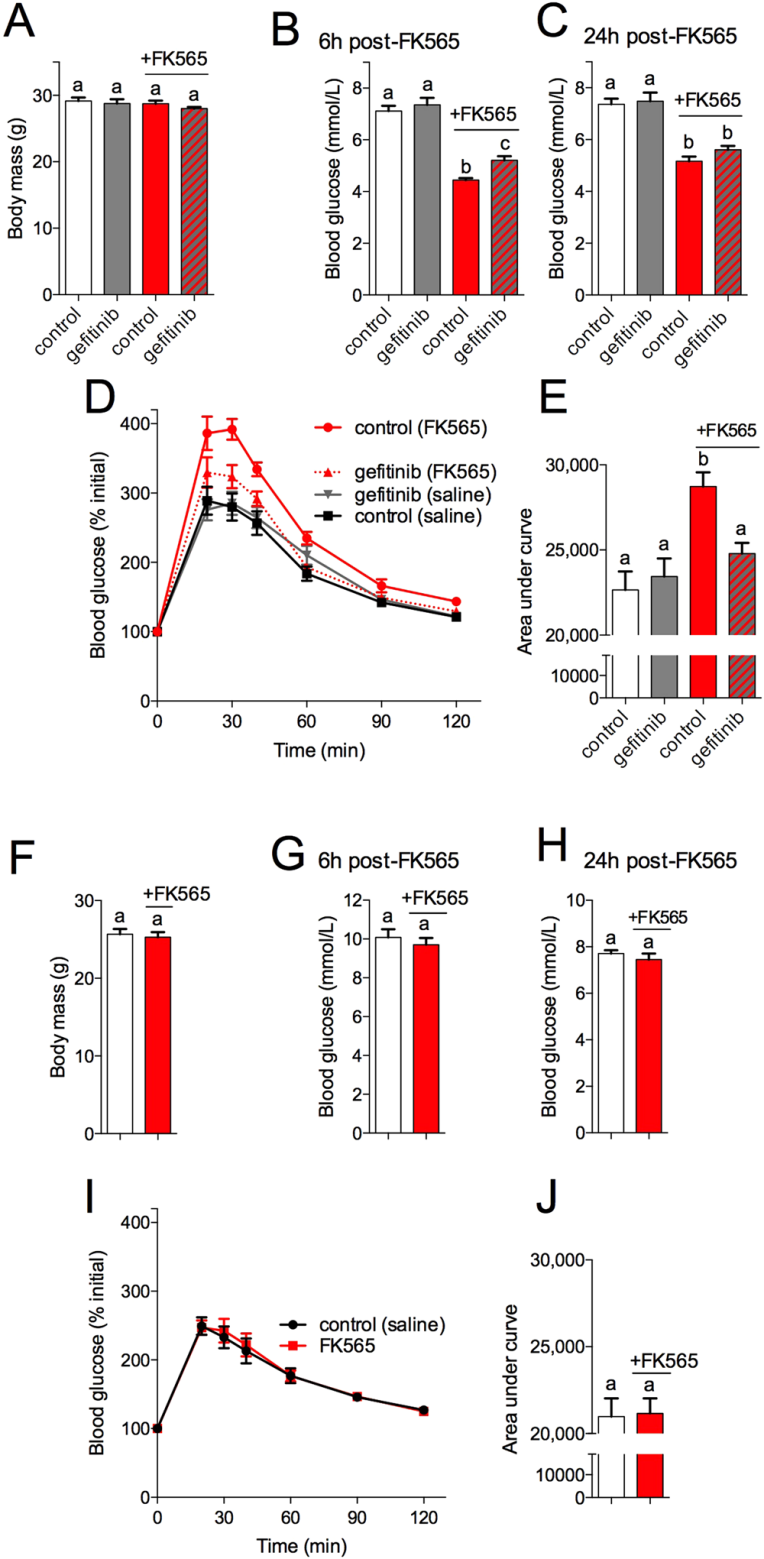
TKI gefitinib inhibits RIPK2-mediated dysglycemia *in vivo*. WT mice pre-treated with TKI gefitinib (100 mg/kg) or methylcellulose vehicle for 4 days before injection of a NOD1 ligand and metabolic testing. Body Mass (**A**) and fasting blood glucose at 6 h (**B**) and 24 h (**C**) after injection of WT mice with FK565 (10 μg, *i*.*p*.) in gefitinib and vehicle pretreated mice, n = 9–12. Percentage change in blood glucose over time (**D**) and area under the curve (**E**) of a glucose tolerance test (2.0 g/kg glucose, *i*.*p*.) 24 h after injection of WT mice with FK565 (10 μg, *i*.*p*.) in gefitinib and vehicle pretreated mice, n = 12. Body Mass (**F**) and fasting blood glucose at 6 h (**G**) and 24 h (**H**) after injection of RIPK2^−/−^ mice with FK565 (10 μg, *i*.*p*.), n = 6–9. Percentage change in blood glucose over time (**I**) and area under the curve (**J**) of a glucose tolerance test (2.0 g/kg glucose, *i*.*p*.) 24 h after injection of RIPK2^−/−^ mice with FK565 (10 μg, *i*.*p*.), n = 9. Values are mean ± SEM. Different letters assigned to each condition (a, b, c) denote statistical differences between groups (p < 0.05).

We previously showed that injection of Nod1 ligands casused acute insulin resistance and dysglycemia in mice^15^. Here, we assessed if gefitinib attenuated dysglycemia induced by Nod1 activation and if this required RIPK2. We chose the lowest dose of gefitinib (100 mg/kg, daily gavage for 4 days) that achieved maximal inhibition of systemic Cxcl1 inhibition *in vivo* to test this hypothesis. Neither gefitinib nor injection of FK565 (10 μg, i.p.) altered body mass (Fig. 7A). Pre-treatment with gefitinib attenuated lower blood glucose at 6 h post-FK565 injection (Fig. 7B). At the time of the glucose tolerance test (GTT, 24 h after FK565 injection) gefitinib pre-treatment did not alter fasting blood glucose (Fig. 7C). However, pre-treatment with gefitinib prevented FK565-induced glucose intolerance during a GTT (Fig. 7D,E). Nod1 activation with FK565 did not alter body mass, fasting blood glucose or glucose tolerance in Ripk2^−/−^ mice (Fig. 7F–J).

## Discussion

Clinical observations of diabetic cancer patients have revealed that TKIs can lower blood glucose despite various growth factor and oncogenic-related targets^2, 3, 5, 29–32^. Understanding off-target effects of existing therapies can mitigate deleterious drug consequences, but it also provides the opportunity to re-task approved drugs^33, 34^. Re-tasking clinically approved TKIs for metabolic disease warrants investigation because a greater understanding of how specific TKIs alter inflammatory and metabolic responses relevant to blood glucose regulation is required to advance the therapeutic potential for different TKIs in metabolic disease. TKIs have a diverse yet overlapping catalog of kinase targets. Understanding the effects of various subsets of TKIs on critical nodes in immunity and metabolism could prioritize the development strategy of these drugs for treatment of metabolic diseases, including diabetes.

TKIs can exert anti-inflammatory effects, and there is a growing interest in expanding the utility of TKIs to combat inflammatory diseases^24, 35–41^. It is plausible that TKI-induced changes in inflammatory status mediate effects on blood glucose regulation and insulin resistance, but it is not yet clear which inflammatory pathways link certain TKIs to changes in metabolic homeostasis. TKIs have multiple off-target effects, including inhibition of Ripk2^25^. For example, TKIs designed to inhibit EGFR signalling have been identified as equipotent Ripk2 inhibitors and TKIs such as ponatinib or sorafenib are even more potent type II inhibitors of Ripk2^23, 24, 42^. This is relevant to obesity-induced metabolic disease because Ripk2 propagates inflammatory signals in response to peptidoglycan and this bacterial cell wall component can promote dysglycemia via Nod1^15^. Here we show that certain TKIs lower Nod1-mediated inflammation and attenuate related metabolic defects. We found that inhibition of Ripk2 was the defining characteristic of TKIs that attenuated peptidoglycan/muropeptide-induced changes in inflammation and metabolism and that Ripk2 was required for Nod1 ligand-induced lipolysis and glucose intolerance in mice.

TKIs can lower blood glucose in some, but not all individuals and mechanisms underlying heterogeneity in patient responses is lacking^43^. Beyond Ripk2, there is evidence for the involvement of other proteins targeted by TKI in pre-diabetes and diabetes, such as c-Abl, PDGFR, EGFR and VEGFR2. It is purported that inhibition of c-Abl can promote pancreatic beta cell survival and enhance insulin production^44–46^. Inhibition of PDGFR may promote adipogenesis and adiponectin secretion while suppressing pancreatic islet inflammation, and VEGFR inhibition reduces T-cell migration and pancreatic insulitis^10, 47–49^. However, a limitation is that only considering a TKIs designed or even most potent kinase target neglects consideration of the multitude of other pathways affected by TKI treatment. For example, treatment of diet-induced obese mice with PD153035 reduces M1 macrophage infiltration into adipose tissue and improves insulin sensitivity^6^. It was hypothesized that inhibition of EGFR (the main target PD153035 is designed against) underlies the observed therapeutic effects. However, we show that PD153035 is an effective inhibitor of Nod1 signalling at relevant concentrations. To abate confounding targets of TKIs, we based the initial experiments on data from a comprehensive analysis of how kinase inhibitors alter the activity of the majority of human kinases^25^, and used a genetic knockout model to determine that our results were specific to Ripk2. We used a reductionist approach to determine if certain TKIs attenuated lipolysis and metabolic tissue inflammation in response to a single microbial-based inflammatory trigger (i.e. peptidoglycan). We directly compared this to another bacterial component (i.e. LPS). This yielded important insight into how TKIs could contribute to metabolic inflammation because only TKIs with a reported inhibitory effect on Ripk2 attenuated inflammation and lipolysis in response to Nod1, but not Tlr4 activating ligands.

**Figure 8:**
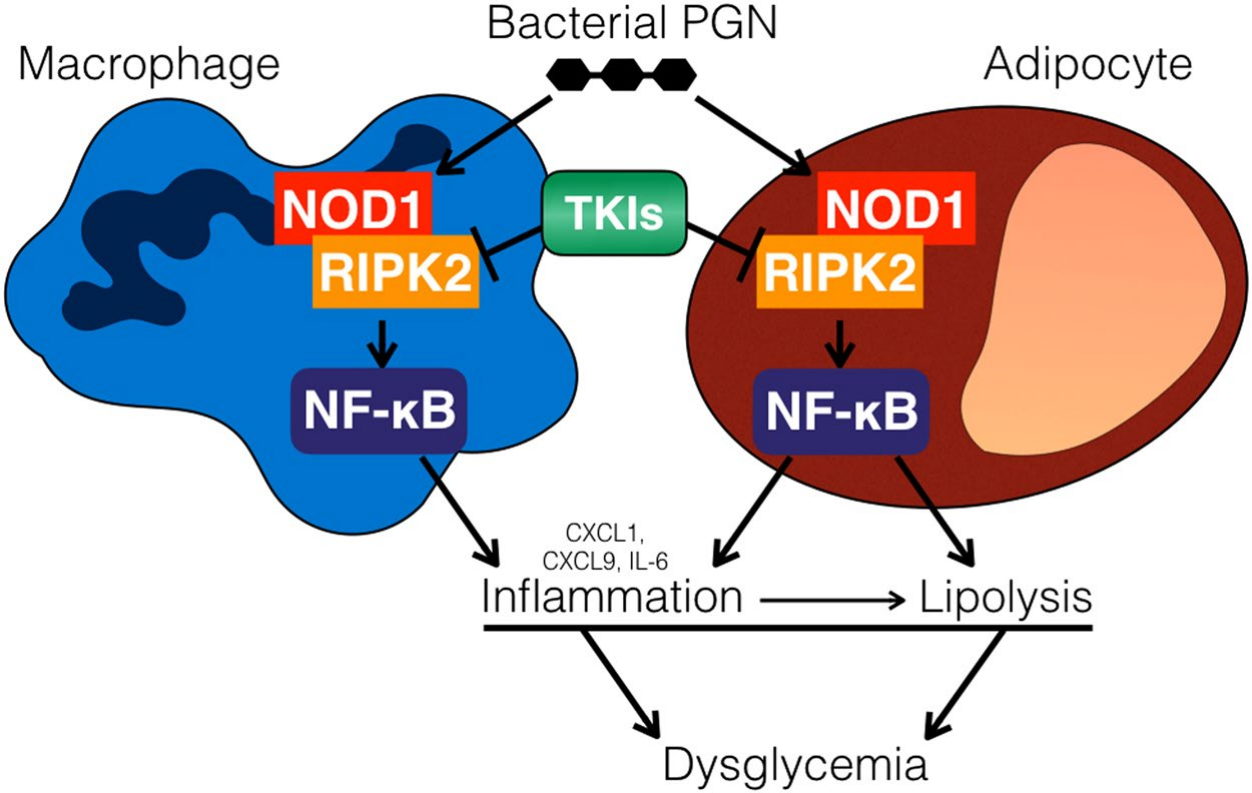
Inflammatory and metabolic effects of TKIs that inhibit RIPK2 in adipocytes and macrophages. RIPK2 is required for increased inflammatory cytokine production and lipolysis induced by bacterial peptidoglycan that acts on NOD1. Certain tyrosine kinase inhibitors (TKIs) inhibit RIPK2 in both macrophages and adipocytes thereby suppressing lipolysis, reducing inflammation and attenuating poor glucose control (dysglycemia) caused by bacterial cell wall components.

It is important to understand how immune pathways underpin TKI-induced changes in glycemia because inflammation can promote insulin resistance and dysglycemia. Our results show that Ripk2 is required for Nod1-mediated inflammation and dysglycemia, and that certain TKIs, such as gefitinib, block bacterial cell wall peptidoglycan-induced inflammation and lipolysis in adipocytes and inflammation in macrophages (Fig. 8). In preliminary experiments, we took advantage of SB203580, an established Ripk2 inhibitor^50^. We show that numerous TKIs are more potent than SB203580 in attenuating Nod1-mediated lipolysis. We also show that SB203580 inhibits both Nod1 and LPS-induced responses in metabolic and immune cells. In contrast, all of our results were consistent with gefitinib attenuating only Nod1-mediated inflammation and lipolysis. These results show the specificity of gefitinib for Nod/Ripk2 responses compared to those for endotoxin.

It is important to consider multiple triggers of inflammation in metabolic disease, including LPS since obesity-related metabolic endotoxemia can promote dysglycemia^17^. When probing the effects of Nod1-mediated dysglycemia, our model demonstrates that activation of Nod1 lowers fasting blood glucose coincident with significant glucose intolerance (i.e. increase in blood glucose levels in response to glucose challenge). The mechanisms for divergent changes in fasting glucose versus glucose tolerance are unknown. However, it is noteworthy that many inflammatory triggers promote insulin resistance and simultaneously lower fasting glucose. Acute, transient decreases fasting blood glucose could occur through increased insulin-independent glucose utilization during inflammation. Nod1-ligand mediated changes in blood glucose are similar to glucose responses observed during acute endotoxemia. Injection of LPS also lowers blood glucose with simultaneously promoting insulin resistance and glucose intolerance^51, 52^. Acute LPS exposure lowers the expression and activity of enzymes involved in glucose production, such as glucose-6-phosphatase (G6Pase) and phosphoenolpyruvate carboxykinase (PEPCK)^53^. Furthermore, NF-κB is induced following LPS exposure, which can further limit glucose production by nitric oxide production and desensitization to glucagon, leading to hypoglycemia^53, 54^. Interestingly, Tumor necrosis factor-α (TNF-α) can also stimulate the same effect as acute LPS exposure, although neutralization of TNF does not completely reverse the metabolic alterations that arise in response to endotoxin challenge^55^. Augmented pro-inflammatory cytokines and MAPK activation induced by inflammatory triggers leads to reduced insulin signalling, which manifests as impaired phosphorylation of IRS-1, Akt and AS160, which reduces insulin-stimulated glucose transport and suppression of lipolyiss^56^. We speculate that many mechanisms underlying Nod1-induced glucose lowering are analogous to those postulated for LPS, including NF-κB activation and overlapping profiles of cytokine release, contributing to alterations in glucose homeostasis. Our results are consistent with this model since a Nod1 ligand activated NF-κB and lowered phosphorylation of Akt in adipocytes, but the Ripk2-inhibitory TKI gefitinib attenuated these Nod1-mediated effects on inflammatory and insulin signalling.

The causes of obesity and diabetes are multifactorial, and there are limitations in applying these findings to metabolic disease. Inflammation can participate in insulin resistance during chronic obesity, but is dispensable for short-term diet-induced insulin resistance^57^. Further, compartmentalization of immune responses in different tissues during obesity is positioned to dictate PAMP or DAMP-related insulin resistance^58^. Therefore, both the duration of obesity and site-specific inflammation may dictate if a certain TKI alters blood glucose. In addition, many triggers of inflammation do not depend on Ripk2. We show here that endotoxin-induced inflammation occurs independently of Ripk2. This is consistent with previous reports showing that Ripk2 mediates Nod1 or Nod2, but not Tlr-induced inflammation^59^. We extend these findings to metabolic cells such as adipocytes and show that gefitinib only inhibits Nod1-ligand, but not Tlr4 ligand-induced inflammatory and metabolic responses.

Even within Ripk2-dependent pathways, we have previously shown divergence in the Nod1 versus Nod2-mediated effects on obesity-related metabolic inflammation and dysglycemia^14, 15^. We have shown here that Ripk2 inhibition by TKIs attenuates Nod1-mediated metabolic defects, but these same TKIs could block potential insulin sensitizing effects of Nod2 activation. It is not yet clear if Nod1 or Nod2 immunometabolism is more important in glucose control during obesity. An important future goal is to understand divergence in Nod1 versus Nod2-induced metabolic effects and how these interact with endotoxemia. Nevertheless, our results have shown that certain TKIs can alter metabolism through Ripk2-mediated responses. The source of the inflammation and Ripk2 should be considered in the multifactorial causes and pathways involved in metabolic inflammation and blood glucose control, which be altered by specific TKIs.

## Materials and Methods

### Mice and materials

All procedures followed the Canadian Council on Animal Care (CCAC) and approved by McMaster University Animal Ethics Review Board (AREB). C57Bl/6J (WT) mice were purchased from JAX (Bar Harbor, ME) and RIPK2^−/−^ mice were kindly provided by Dr. Richard Flavell and supplied by Dr. Dana Philpott. FK565 (heptanoyl-γ-D-glutamyl-L-*meso*-diamino-pimelyl-D-alanine) was obtained from Astellas Pharma (Tokyo, Japan). Ultra pure lipopolysaccharide (LPS; tlrl-ppglps), Normocin^TM^ (ant-nr-1), Blasticidin^TM^ (ant-bl-1), Zeocin^TM^ (ant-zn-1), HEK-Blue^TM^ Detection media (hb-det3), HEK-Blue^TM^ mNOD1 (hkb-mnod1) cells and HEK-Blue^TM^ mTLR4 (hkb-mtlr4) cells were obtained from Invivogen (San Diego, CA). Gefitinib and erlotinib were from AbMole Bioscience (Houston, TX). SB203580, Compound 56, neratinib, lapatinib, and PD153035 hydrochloride were from ApexBio (Houston, TX). Tyrphostin AG 1478 was from Cell Signalling Technology (Boston, MA). Ponatinib, dasatinib, dabrafenib, vandetanib, ruxolitinib, ibrutinib sorafenib, crizotinib, imatinib and sunitinib were from AdooQ Bioscience (Irvine, CA). Methylcellulose (M0512), isoproterenol (I6504), (-)-N^6^-(2-phenylisopropyl)adenosine (PIA; P4532) and fatty acid-free bovine serum albumin (BSA) were from Sigma-Aldrich (St. Louis, MO). GlutaMAX, Dulbecco’s modified Eagle medium (DMEM), Dulbecco’s phosphate-buffered saline (DPBS) and fetal bovine serum (FBS) were from Life Technologies (Burlington, ON).

### Cell culture

Murine 3T3-L1 preadipocytes (American Type Culture Collection, Rockville, MD) were cultured in DMEM containing 10% FBS, 1% GlutaMAX and 1% penicillin-streptomycin (complete culture medium). When cells reached 80% confluence, differentiation was induced by incubating with complete culture medium containing 0.5 mM 3-isobutyl-1methylxanthine, 0.25 μM dexamethasone, 10 μg/mL insulin and 2 μM rosiglitazone for 48 hours (day 0 to 2) and then changed to complete culture medium containing only 10 μg/mL insulin for 48 hours (day 2 to 4). Complete culture medium containing only 10 μg/mL insulin was replaced every 48 hours and experiments were performed on adipocytes he differentiated for 8–12 days. Bone marrow derived macrophage (BMDM) cells were harvested from femur and tibia of C57Bl/6J (WT) or RIPK2^−/−^ mice and cultured for 7–10 days in DMEM containing 10% FBS and 15% L929 conditioned media, as described^60^. On day 4, media was replenished with additional 15% L929 conditioned media. Prior to treatment, adipocytes were washed twice with warmed DPBS and maintained in serum-free DMEM containing 0.5% fatty-acid free BSA and 1% pen/strep (treatment media). BMDM cells were washed twice with warmed DPBS and maintained in DMEM containing 0.5% FBS and 1% pen/strep. Adipocytes and BMDM cells were pre-incubated for 1 h with inhibitors and subsequently treated with or without 10 μg/mL FK565 or 500 ng/mL LPS. Glycerol concentrations were determined using a free glycerol determination kit from Sigma-Aldrich (St. Louis, MO). Cxcl1 and Il6 were quantified by ELISA from R&D Systems (Denver, CO). For insulin signalling, following 1 h pre-incubation with 5 μM gefitinib and 48 hour treatment with 10 μg/mL FK565, adipocytes were stimulated for 10 minutes with 100 nM insulin and washed twice in ice-cold PBS. Cell lysates were prepared and immunoblotting were performed as previously described^15, 60^. For transcript analysis, total RNA was prepared using Trizol (Invitrogen; Carlsbad, CA) and qPCR was performed as previously described^14^ using the Rotorgene 6000 (Qiagen; Toronto, ON). HEK-Blue™ NOD1 cells were grown in media containing DMEM, 10% FBS, 1% Glutamine, 1% p/s, 100 µg/mL Normocin^TM^, 30 µg/mL Blasticidin^TM^ and 10 µg/mL Zeocin^TM^ (HEK-NOD1 media). Cells were seeded at a density of 30,000 cells/well into a 96-well plate and incubated for 24 hours prior to treatment. At time of cell treatment, cells were switched into HEK-Blue™ detection media supplemented with 1 or 5 µM gefitinib, as appropriate. Cells were incubated for 1 hour with or without gefitinib, before stimulation with 10 µg/mL FK565 or 0.5 µg/mL LPS. 24 hours later, the secreted alkaline phosphatase (SEAP) reporter of NF-κB activity was detected by measuring absorbance at 630 nM on a Synergy H4, Hybrid Reader (BioTek, Winooski, VT). All cells were maintained at 37 °C and 5% CO_2._

### Animal protocols

C57Bl/6J (WT) and RIPK2^−/−^ mice were maintained under controlled lighting (12:12 L;D) and temperature (22 °C) with *ad libitum* access to standard chow diet and water. Gefitinib was administered for 4 days, across a range of doses (5–200 mg/kg/day) by oral gavage. For all experiments, the dose of gefitinib was based on the body weight measured on Day 1 and Day 3. Gefitinib was suspended in 1% methylcellulose at 1.25–50 mg/mL with brief sonication followed by thorough vortexing to achieve a homogenous colloid and animals were gavaged with 130–190 μL based on body weight. Following four consecutive days of gefitinib administration, mice were injected with Nod1 ligand FK565 (10 μg, i.p.). Blood samples were collected via tail-vein sampling at t = 0, 2 and 6 h post-injection. Blood was incubated at RT for 20 min, centrifuged for 5 min at 4 °C and 7500 rpm, blood serum was collected and stored at −80 °C. After 6 h blood samples were collected, mice were euthanized by cervical dislocation and gonadal adipose tissue depots were rapidly excised and snap frozen in liquid nitrogen. In adipose, transcript analysis was performed as previously described^14^ and Cxcl9 was quantified by ELISA from R&D Systems (Denver, CO). For GTTs, animals were orally gavaged with gefitinib (100 mg/kg/d) for 4 days as described above, and injected with FK565 (10 μg, i.p.) on day 4. 24 h later, a GTT was performed in 6 h fasted mice. Mice were injected with glucose (2.0 g/kg, *i*.*p*.) and blood glucose was repeatedly measured via tail vein sampling using an Accu-Chek Aviva blood glucometer from Roche Diagnostics (Mississauga, ON). Area under the curve (AUC) of blood glucose was calculated using GraphPad Prism 4–6 software.

### Data analysis

Data is expressed as mean ± standard error of the mean (SEM). Comparisons were made using unpaired, two-tailed Student’s t-test, where 2 variables are compared. ANOVA, was used for comparison of more than 2 variables and Tukey’s post hoc test was used when appropriate (Prism 4–6; Graphpad Software).

### Data Availability

The datasets generated during and analysed during the current study are available from the corresponding author on reasonable request.

## Electronic supplementary material

Figures S1-S3
