# Supplementary material for: Tyrosine kinase inhibitors of Ripk2 attenuate bacterial cell wall-mediated lipolysis, inflammation and dysglycemia
Source: Sci Rep. 2017 May 8;7:1578. doi: 10.1038/s41598-017-01822-0 (PMC5431485; doi:10.1038/s41598-017-01822-0)
Supplement: Supplementary file 1 — Figures S1-S3 [file 41598_2017_1822_MOESM1_ESM.pdf]

Supplementary Information

Tyrosine kinase inhibitors of Ripk2 attenuate bacterial cell wall-mediated lipolysis, inflammation and dysglycemia

Authors: Brittany M. Duggan, Kevin P. Foley, Brandyn D. Henriksbo, Joseph F. Cavallari, Akhilesh K. Tamrakar, Jonathan D. Schertzer.

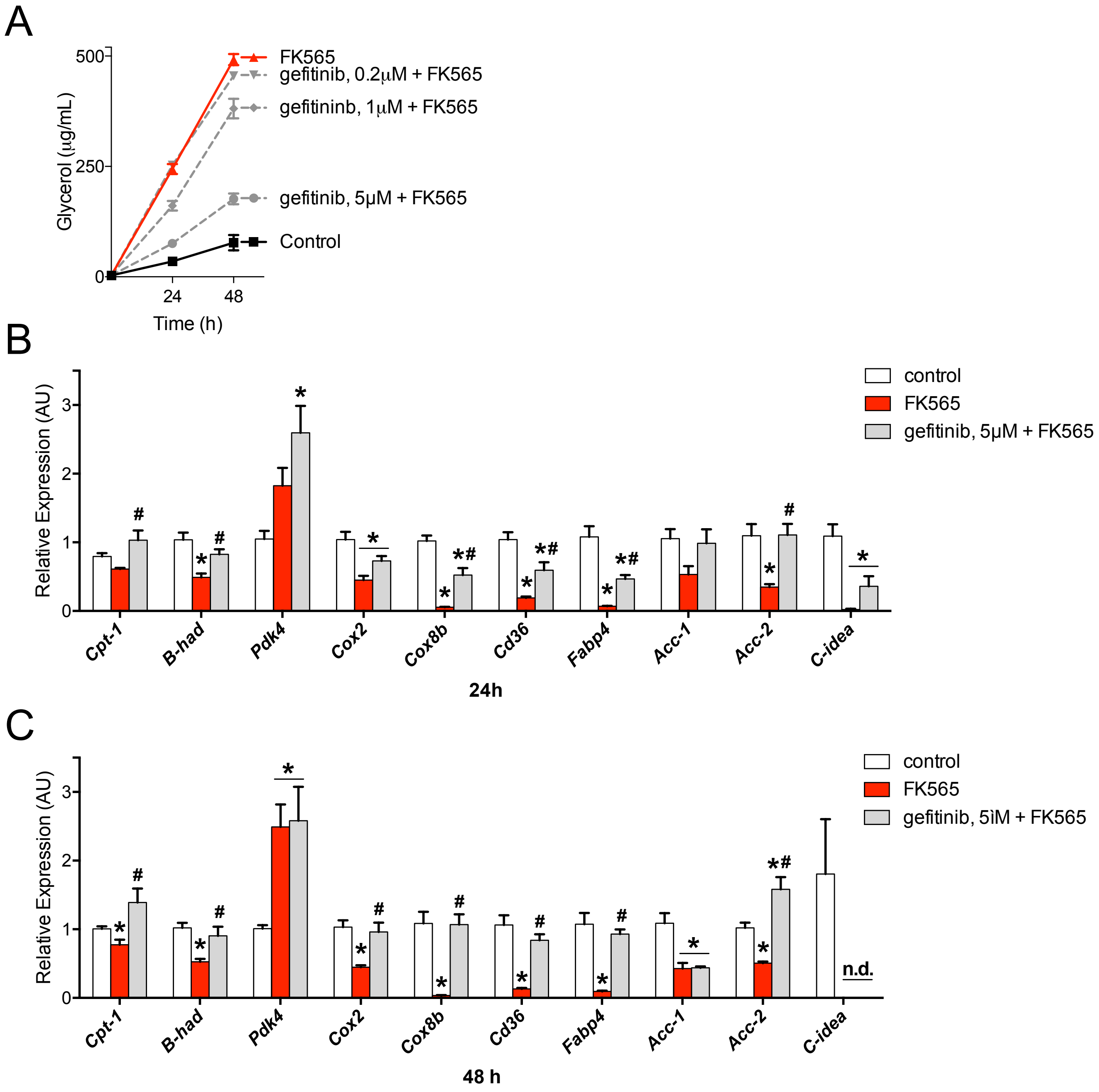

Supplementary Figure 1. Time course of glycerol release in 3T3-L1 adipocytes pre-incubated with gefitinib and stimulated with the NOD1 ligand FK565 (10µg/mL), n=4-12 (A). Changes in gene expression of molecular markers of lipid oxidation, transport and synthesis 24 h (B) and 48 h (C) after stimulation with the NOD1 ligand FK565 (10µg/mL) in 3T3-L1 adipocytes pre-incubated with gefitinib, n=7-8. Values are mean $\pm$ SEM. * denotes statistically different from Control condition, # denotes statistically different from FK565 condition ($p<0.05$).

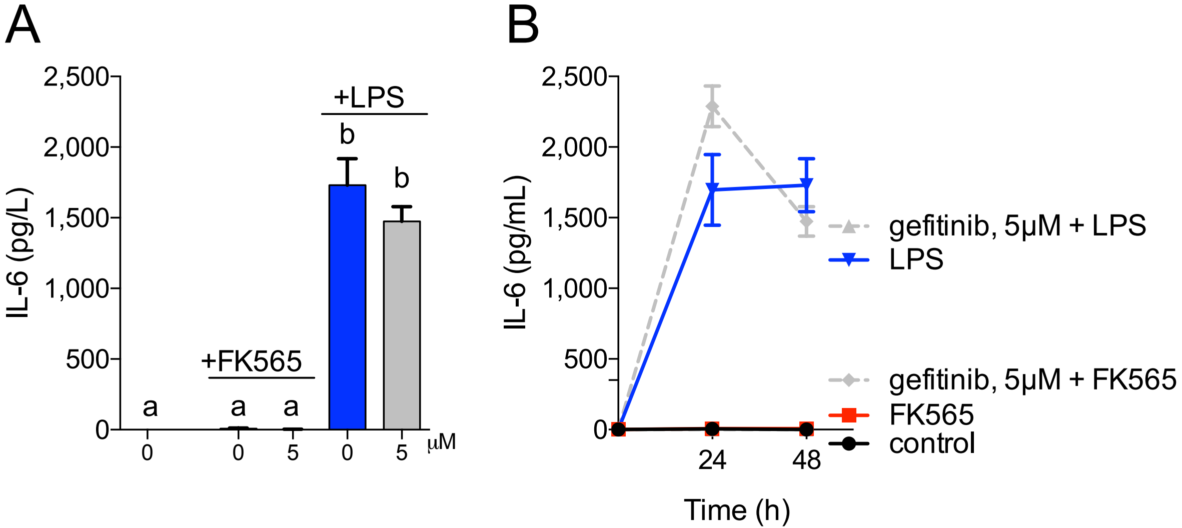

Supplementary Figure 2. Levels of Il-6 secreted from Bone marrow-derived macrophages (BMDMs) are below the detectable level 48 hours after stimulation with the NOD1 ligand FK565 (10µg/mL). Levels of IL-6 secreted from BMDMs are stimulation with the Tlr4 ligand LPS (0.5 µg/mL) pre-incubated for 1h with gefitinib (A), n=5. Time course of FK565-stimulated or LPS-stimulated Il-6 secretion (B), n=5. Values are mean mean + SEM. Different letters assigned to each condition (a, b, c) denote statistical differences between groups ($p<0.05$).

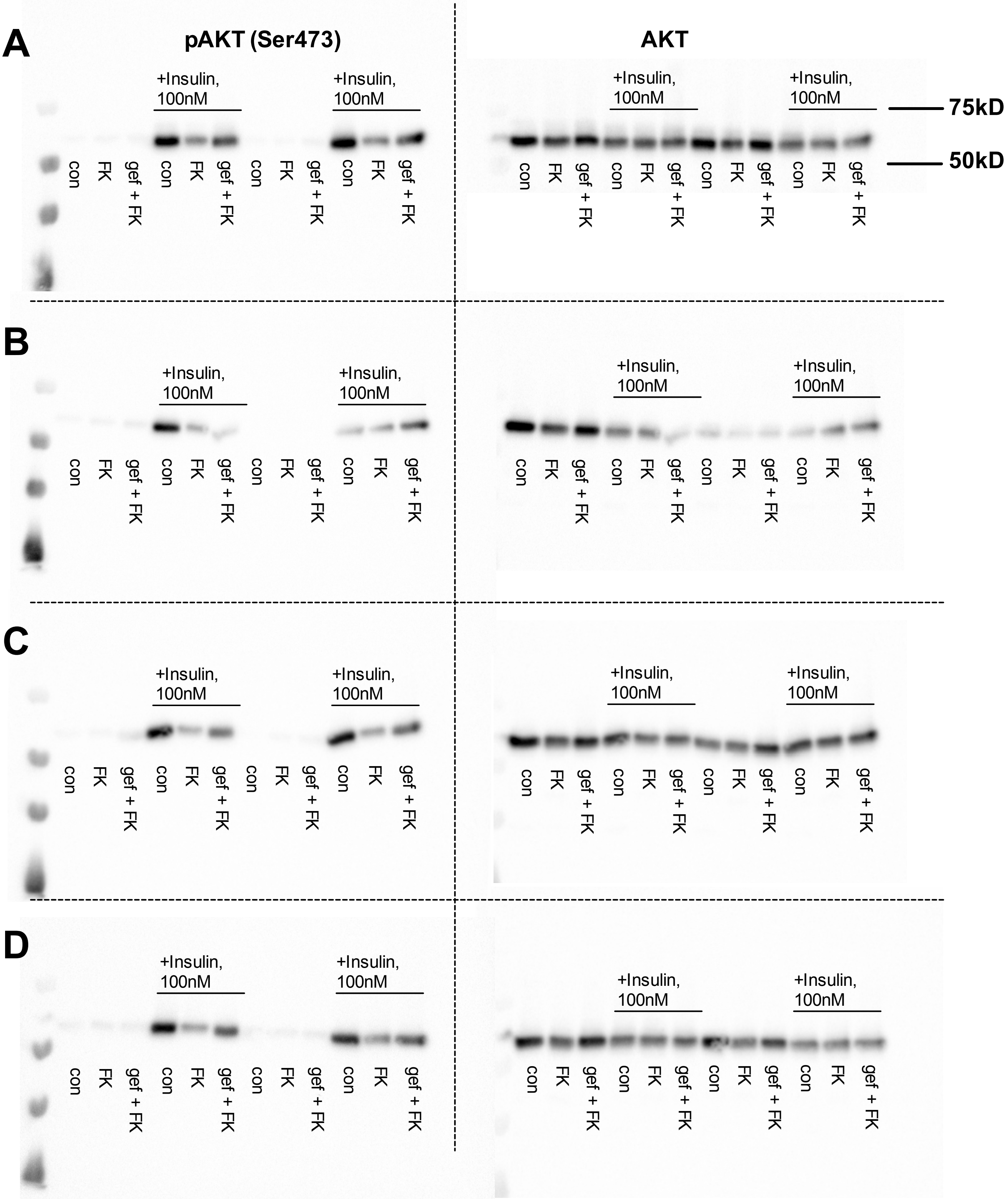

Supplementary Figure 3. Immunoblots of basal (ie. no insulin) and 100nM insulin-stimulated pAKT (Ser473; left panel), and total AKT (right panel) from 4 different blots (A-D). For each condition, n=2 basal and n=2 insulin-stimulated samples were included on each gel. 'Con' = Control, 'FK' = FK565 (10µg/mL) and 'gef +FK' = gefitinib (5µM) + FK565 (10µg/mL) Quantitative comparison was conducted between samples from all 4 blots, derived from the experiment and processed in parallel.
